# Ethical implications of ChatGPT and other large language models in academia

**DOI:** 10.3389/frai.2025.1615761

**Published:** 2025-09-01

**Authors:** Ahmad Almufarreh, Ashfaq Ahmad, Muhammad Arshad, Choo Wou Onn, Robinson Elechi

**Affiliations:** ^1^Deanship of Human Resources and Information Technology, Jazan University, Jazan, Saudi Arabia; ^2^Faculty of Basic Sciences, Lahore Garrison University, Lahore, Pakistan; ^3^School of Informatics and Cybersecurity, Technological University Dublin, Dublin, Ireland; ^4^UNICAF, Larnaca, Cyprus; ^5^Faculty of Data Science and Information Technology, INTI International University, Nilai, Malaysia; ^6^University of East London, London, United Kingdom

**Keywords:** quality education, artificial intelligence, ChatGPT, emerging technologies in education, large language models

## Abstract

The rapid advancement of technology in the digital age has significantly transformed human communication and knowledge exchange. At the forefront of this transformation are Large Language Models (LLMs), powerful neural networks trained on vast text corpora to perform a wide range of Natural Language Processing (NLP) tasks. While LLMs offer promising benefits such as enhanced productivity and human-like text generation, their integration into academic settings raises pressing ethical concerns. This study investigates the ethical dimensions surrounding the use of LLMs in academia, driven by their increasing prevalence and the need for responsible adoption. A mixed-methods approach was employed, combining surveys, semi-structured interviews, and focus groups with key stakeholders, including students, faculty, administrators, and AI developers. The findings reveal a high level of LLM adoption accompanied by concerns related to plagiarism, bias, authenticity, and academic integrity. In response, the study proposes concrete strategies for ethical integration, including: (1) the establishment of transparent usage policies, (2) the incorporation of LLM literacy training into academic curricula, (3) the development of institutional review frameworks for AI-generated content, and (4) ongoing stakeholder dialogue to adapt policies as the technology evolves. These recommendations aim to support the responsible and informed use of LLMs in scholarly environments. The widespread influence of technological advancement has notably transformed communication and knowledge sharing, with LLMs playing a central role. These advanced neural networks, trained on extensive text datasets, have become valuable tools for generating human-like text and improving efficiency. However, their growing use in academic contexts raises significant ethical concerns. This study investigates these issues, focusing on the implications of LLM integration in scholarly environments. Using mixed methods, including surveys, semi-structured interviews, and focus groups, the research gathered insights from students, faculty, administrators, and AI developers. The findings highlight substantial adoption of LLMs alongside concerns about plagiarism, bias, and academic integrity. Based on this input, the study proposes guidelines for their responsible and ethical use in academia.

## 1 Introduction

The exponential growth of AI tools, particularly Large Language Models (LLMs) like OpenAI’s ChatGPT and Google’s LaMDA, is revolutionizing education, healthcare, and industry. However, their integration into academia raises profound ethical questions surrounding authorship, bias, and academic integrity. This paper investigates the ethical implications of LLM use in academic settings by exploring stakeholder perceptions and developing practical guidelines for responsible integration.

The emergence of Large Language Models (LLMs) like OpenAI’s GPT series marks a significant shift in the digital landscape, transforming sectors such as education, healthcare, and engineering. Models like GPT-3, GPT-4, Google’s LaMDA (Bard), and Microsoft’s GPT-3 offer human-like text generation, but their rise presents ethical challenges. In education, LLMs, including ChatGPT, raise concerns about authorship, bias, privacy, and the integrity of academic standards. While LLMs enhance productivity by providing quick, high-quality content, they may undermine students’ learning by reducing opportunities for critical thinking and authentic expression. Key ethical issues include:

- *Authorship and ownership*: LLMs challenge traditional notions of intellectual ownership, raising questions about proper attribution and originality.
- *Bias and fairness*: AI-generated content may perpetuate harmful biases embedded within training datasets.
- *Privacy and consent*: LLMs trained on vast, often unregulated data sources introduce significant privacy risks.

Although LLMs offer convenience, their unchecked use in academia risks compromising the educational system’s goals of developing critical skills and fostering innovation. Therefore, integrating these tools requires careful ethical consideration to ensure alignment with the core principles of education and responsible use of AI.

## 2 Literature review

The rapid integration of Large Language Models (LLMs) like ChatGPT into academia has sparked significant debate regarding their ethical implications. Existing research highlights various challenges, including concerns about authorship, bias, privacy, and the broader impact on teaching and learning. This section critically reviews the literature, organized thematically to provide a coherent and comparative understanding of these ethical challenges.

### 2.1 Authorship and intellectual ownership

The question of authorship and intellectual ownership has emerged as a primary ethical concern in the age of LLMs. Stokel-Walker (2023) emphasizes the need to redefine intellectual contributions, warning that traditional notions of authorship risk becoming obsolete as AI-generated content becomes prevalent.

Similarly, Lund and Wang (2023) discuss the ethical dilemmas LLMs pose to academic integrity, particularly regarding unclear attribution of AI-assisted work. However, while these studies underline the problem, Anderson and Dill (2000) highlight a deeper issue: without strict criteria, human contributions can be underestimated, and distinguishing AI-generated content from human work becomes increasingly challenging. This highlights the urgent need for clear attribution guidelines to preserve academic integrity and ensure human intellectual efforts are properly recognized.

### 2.2 Bias and fairness in LLM outputs

A consistent thread in the literature concerns the biases inherent in LLM outputs. Bender et al. (2021) demonstrate how LLMs can perpetuate harmful social biases embedded in their training data, posing risks to fairness and inclusivity in academia. Nadeem et al. (2021) comparative study of gender bias across cultural datasets further illustrates that these biases are not only technical flaws but also reflect broader societal inequities. To address these challenges, Sun et al. (2019) propose mitigation strategies such as Counterfactual Data Augmentation and Self-Debiasing. While these approaches show promise, the persistence of cultural and gender biases across datasets, as reported by Nadeem et al. (2021), suggests that technical solutions alone are insufficient. A more holistic, ethically grounded approach to LLM development and deployment is required to prevent these technologies from amplifying existing social inequalities in educational settings.

### 2.3 Privacy and data security concerns

The widespread use of LLMs in academia has also heightened concerns about privacy and data security. Diakopoulos (2016) argues that LLMs’ reliance on large, often unregulated datasets introduces significant risks to user privacy. This concern has been echoed in regulatory responses such as Italy’s temporary ban on ChatGPT, highlighted by Vinuesa et al. (2020), which reflects growing international calls for accountability in AI systems. While some scholars suggest that the benefits of LLMs may justify limited trade-offs in privacy (Vinuesa et al., 2020), others emphasize the importance of transparency and stringent data governance to maintain trust in educational institutions. Without proper safeguards, the integration of LLMs into academia may compromise not only individual privacy but also institutional credibility.

### 2.4 Pedagogical impacts and student engagement

LLMs offer both opportunities and risks for pedagogy and student engagement. Holmes et al. (2019) describe the potential of AI technologies to personalize learning and enhance educational methods. Wang and Akhter (2025) explored how emotional and behavioral regulation strategies influence student learning outcomes when using different AI tools, finding that ChatGPT users exhibited more consistent vocabulary retention and lower levels of hopelessness compared to those using Bing’s AI interface. However, Schölkopf and Vapnik (2002) caution that over-reliance on such technologies may undermine the development of critical thinking skills, a core objective of higher education. From a social perspective, Alshahrani et al. (2024) argue for the responsible use of AI to foster meaningful student engagement. This perspective aligns with recent systematic reviews and case studies highlighting that, when properly integrated, tools like ChatGPT can enhance student engagement and promote deeper learning by providing personalized feedback and improving access to information (Bettayeb et al., 2024; Khurma et al., 2024). Recent evaluations of LLMs’ performance on clinical reasoning tasks further illustrate their potential for high-stakes educational settings. For instance, Shieh et al. (2024) assessed ChatGPT-4.0’s capabilities on the USMLE Step 2 CK and found that it demonstrated diagnostic reasoning skills comparable to medical trainees, reinforcing the model’s utility in structured academic assessments. However, this reinforces the need for well-defined boundaries and context-specific guidelines to prevent misuse. However, these studies also emphasize that the potential benefits are contingent upon clear institutional policies that mitigate misuse, address ethical concerns, and ensure alignment with educational objectives.

### 2.5 Regulation and responsible AI use

There is a broad consensus in the literature on the need for robust AI regulations to safeguard educational integrity. Crompton and Burke (2023) underscores the importance of clear policies addressing privacy, transparency, and authenticity in AI use. Similarly, the E. Commission’s (2021) proposed Artificial Intelligence Act emphasizes independent audits for high-risk AI systems, including those used in education. While Chatterjee (2020) and Tartaro et al. (2023) acknowledge that regulations can slow innovation, Castro and McLaughlin (2019) advocate for a balanced approach that prioritizes both safety and technological progress. This balance is particularly critical in academia, where innovation must not compromise ethical standards or institutional credibility. Mackrael (2023) further notes that the European Union’s draft AI laws represent an important step toward achieving this balance. Interestingly, Madkhali and Sithole (2023) highlight how AI integration in education can align with broader societal goals, such as sustainability, as seen in Saudi Arabia’s practices. This suggests that, beyond compliance, AI policies can contribute to larger educational and social objectives when implemented thoughtfully. Arshad et al. (2025) emphasize that advancements in AI-driven analysis—such as forensic tools for social media data—demonstrate the critical need for ethical oversight when deploying intelligent systems in sensitive domains like education or criminal justice.

### 2.6 Academic integrity and detection of AI-generated work

The challenge of academic dishonesty fueled by LLMs is well-documented. Rudolph et al. (2023) reports that concerns about plagiarism have led some academic institutions to restrict ChatGPT use altogether. While OpenAI’s AI Classifier and similar tools were developed to detect AI-generated content, studies by Crothers et al. (2023) reveal that these tools often suffer from low accuracy, making detection unreliable. Efforts to develop more effective techniques, such as watermarking and machine learning-based detection, are ongoing (Crothers et al., 2023). Additionally, universities are exploring data mining and AI applications within student workflows, such as admissions processes (Schölkopf and Vapnik, 2002), though these innovations carry their ethical complexities. Additionally, Ramoni et al. (2024) explore how LLMs are being used to generate scientific medical writing, cautioning against both legitimate and deceptive practices. Their findings underline the risk that AI-generated manuscripts may blur ethical lines in authorship and originality if detection systems and citation protocols are lacking.

### 2.7 Detection of AI-generated content and academic integrity

The detection of AI-generated content remains a major concern for maintaining academic integrity. Kirchenbauer et al. (2023) highlight the emerging role of watermarking techniques to distinguish AI-generated outputs from human work, presenting them as a potential solution to uphold academic standards. However, the effectiveness and practicality of watermarking in dynamic educational environments continue to be debated. Similarly, recent advancements in machine learning and data mining are reshaping academic workflows beyond detection. Assiri et al. (2024) demonstrate how universities are leveraging these technologies to enhance student admission procedures, improving efficiency and decision-making. While promising, these developments raise further ethical questions regarding transparency and bias in automated academic processes.

### 2.8 AI usage trends and institutional responses

AI adoption in academia is accelerating. Saúde et al. (2024) reports that the growing use of AI tools like ChatGPT is reshaping academic practices, though institutions face significant challenges in policy development and ethical oversight. Supporting this, Saúde et al. (2024) emphasizes the need for proactive educational strategies to ensure students are not disadvantaged by the rapid technological shift. Some institutions have already begun integrating ChatGPT into curricula to enhance digital literacy and student preparedness (Saúde et al., 2024). Media reports further indicate that schools worldwide are introducing AI education to prevent students from falling behind (Saúde et al., 2024).

### 2.9 Broader implications of AI in education and other sectors

While LLMs dominate discussions around AI in education, broader technological advancements such as deep learning and the Internet of Things are also transforming adjacent sectors. Refaee and Shamsudheen (2022) demonstrate how these technologies are driving improvements in healthcare systems, offering insights into the responsible integration of AI in high-stakes domains, including education. Assessing learning environments is another crucial aspect of responsible AI adoption. Hasan and Gupta (2013) highlight that understanding students’ learning contexts is vital for fostering academic success. These insights are directly applicable to AI integration in educational settings, underscoring the need to align technological tools with conducive learning environments. Arshad et al. (2025) highlight that the integration of big data analytics and AI in digital platforms demonstrates how algorithmic technologies can enhance personalization and decision-making efficiency, principles that are increasingly relevant for AI adoption in academic settings. From a benchmarking perspective, Tordjman et al. (2025) present an extensive evaluation of the DeepSeek model in medical contexts, showing its competitive clinical reasoning abilities across various diagnostic tasks. This comparative analysis not only highlights LLMs’ evolving strengths but also raises questions about academic standards and the readiness of institutions to assess AI contributions in specialized disciplines.

### 2.10 Summary of literature gaps

Despite extensive discourse, the literature reveals key gaps in understanding the lived experiences and perspectives of academic stakeholders regarding LLM use. While existing studies identify technical risks and propose mitigation strategies, there remains a lack of empirical research examining how students, faculty, and administrators perceive these technologies in academic contexts. This study addresses that gap by providing data-driven insights into stakeholder perceptions, contributing to a more holistic understanding of the ethical dimensions of LLM integration in academia.

While existing studies have highlighted the potential ethical risks associated with LLMs, including plagiarism, bias, and data privacy issues (Anderson and Dill, 2000; Bender et al., 2021), this study builds on this body of work by examining how different academic stakeholders perceive these challenges. By comparing the experiences and concerns of students, faculty, and administrators, this study offers new insight into the specific ethical dilemmas faced in academic settings.

Our findings confirm that plagiarism remains a central concern, as evidenced by 70.73% of respondents associating LLMs with accidental plagiarism. However, this study also challenges some previous assumptions about AI’s role in academia. For instance, while earlier literature suggested that AI technologies could potentially diminish critical thinking (Schölkopf and Vapnik, 2002), our data show a mixed perception among respondents. Some stakeholders view LLMs as tools that can enhance productivity without compromising learning, while others express concerns that over-reliance on such technologies could undermine academic integrity.

These findings extend the work of scholars like Sun et al. (2019) and Nadeem et al. (2021), who have explored bias in AI-generated content. Our research not only reaffirms the significance of this issue but also provides a nuanced understanding of how these biases manifest in academic contexts. For example, our respondents indicated that AI tools can amplify existing biases, especially in areas like gender and cultural representation. This underscores the need for more comprehensive measures to mitigate these biases in educational settings.

## 3 Research methodology

This section outlines the methods and strategies employed to achieve the research objectives, with a focus on data collection, stakeholder analysis, and ethical considerations for deploying large language models (LLMs) in education. An interpretive approach is adopted, emphasizing the development of guidelines from collected data rather than pre-existing theories.

### 3.1 Sampling

Due to practical constraints, a non-probability sampling approach is used, including:

- *Purposeful sampling*: Selecting participants with relevant characteristics to ensure diverse academic perspectives.
- *Snowball sampling*: Relying on initial participants to recommend additional experts.
- *Sample size and validation*: Aiming for statistical robustness while considering resource constraints. Participant demographics (e.g., age, gender, and affiliation) will be reviewed to ensure sample representativeness.
- *Informed consent*: Participants will be informed of the study’s goals and ethical practices.

While a non-probabilistic sampling approach was employed due to practical constraints, it is important to note that this choice limits the generalizability of the findings to the broader academic population. The study aimed for a targeted sample that represented key academic stakeholders (students, faculty, administrators, and developers). However, the lack of randomness in sample selection means the results may not fully reflect the diversity of experiences across all academic institutions. We acknowledge this limitation and caution against overgeneralizing the findings to larger populations. The final sample size was 41 respondents, which is appropriate for qualitative insights but may not provide the statistical power needed for broader generalization.

To mitigate the inherent bias of non-probabilistic sampling, we employed purposeful and snowball sampling techniques. These approaches allowed us to gather insights from participants who have direct experience or interest in the ethical implications of LLMs. However, the limitations of these methods, such as the potential for homogeneity in responses, were considered when interpreting the results.

### 3.2 Data collection method

A mixed methods approach will combine both quantitative and qualitative data. The data collection process took place from June 5 to November 19, 2023. During this timeframe, responses were gathered from participants via a combination of surveys and semi-structured interviews.

- *Surveys*: Distributed via Google Forms to assess perceptions of ethical challenges associated with LLMs across various stakeholders.
- *Semi-structured interviews*: Conducted through Zoom to gather deeper insights into participants’ experiences and recommendations.
- *Focus groups*: Facilitating collaborative discussions to explore diverse viewpoints, though peer influence is acknowledged as a potential limitation.

### 3.3 Data analysis

This phase involves a rigorous examination to derive meaningful insights and formulate ethical guidelines.

1. *Quantitative analysis*:

- *Descriptive statistics*: Calculating key metrics (e.g., mean, median) to summarize survey responses.
- *Correlation and regression analysis*: Exploring relationships and predictive factors, such as the link between demographic variables and concerns about LLM biases.

2. Qualitative analysis:

- *Thematic analysis:* Identifying recurring themes related to bias, privacy, and academic integrity.

3. *Scoring*: Converting qualitative insights into numerical values to enhance statistical analysis.
4. *Sentiment analysis*: Assessing opinions using Python libraries to determine whether sentiments are positive, neutral, or negative.
5. *Reporting and interpretation*: Results will be presented through tables, charts, and narrative descriptions, aligning findings with research goals. Data triangulation will ensure reliability by cross-referencing different sources, while strict ethical measures will protect participants’ confidentiality and anonymity.

To ensure the validity and reliability of the study, several measures were implemented. Triangulation was used to cross-check findings across multiple data sources (surveys, interviews, and focus groups). Additionally, inter-rater reliability was conducted to ensure consistency in the qualitative analysis, particularly during thematic analysis. This rigorous approach to data analysis contributes to the trustworthiness of the results.

## 4 Results and findings

This section presents the findings of our study on the ethical considerations of LLMs in academia, exploring both their benefits and challenges. It methodically presents data from surveys, interviews, and discussions, reserving interpretations for the next chapter. It aims to maintain relevance and conciseness, adhering to ethical reporting standards and safeguarding participant anonymity.

### 4.1 Data preparation

Before analysis, the survey data underwent preparatory organization to suit the analytical methods, ensuring alignment with the analysis requirements. Adjustments were made to categorize respondents under single roles, prioritizing their primary responsibilities, and ambiguous responses were recorded to maintain clarity and consistency in the dataset.

### 4.2 Participants

**Figure 1:**
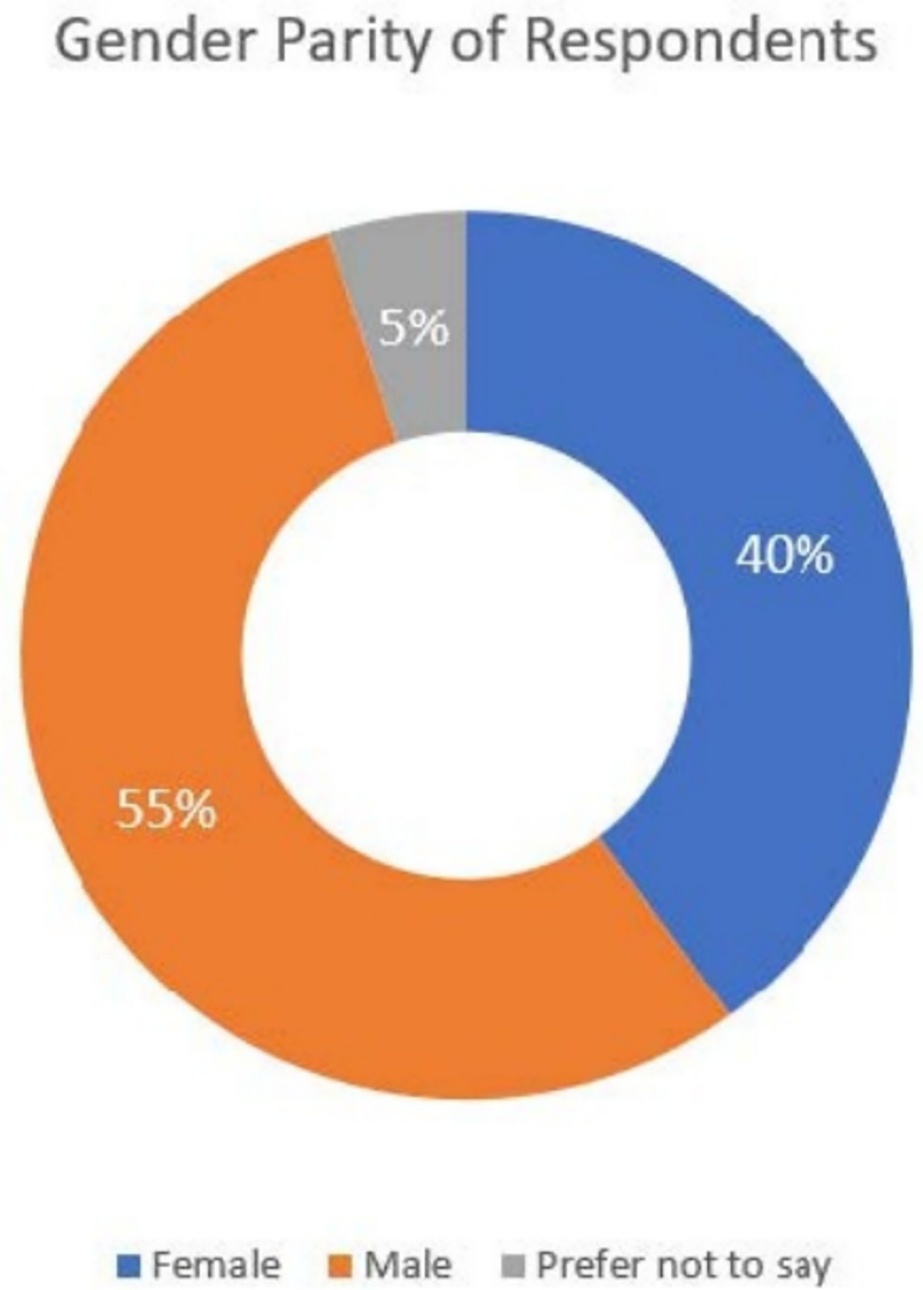
Gender parity of respondents.

There were a total of 41 respondents, with a gender distribution of 55% male and 40% female, as illustrated in Figure 1. The remaining 5% preferred not to disclose their gender.

Among the survey respondents, the largest group consisted of undergraduate computer science students (41.46%) in their final year of study. The teaching staff, which included all faculty members in colleges, universities, and schools, represented 24.39% of the participants.

**Figure 2:**
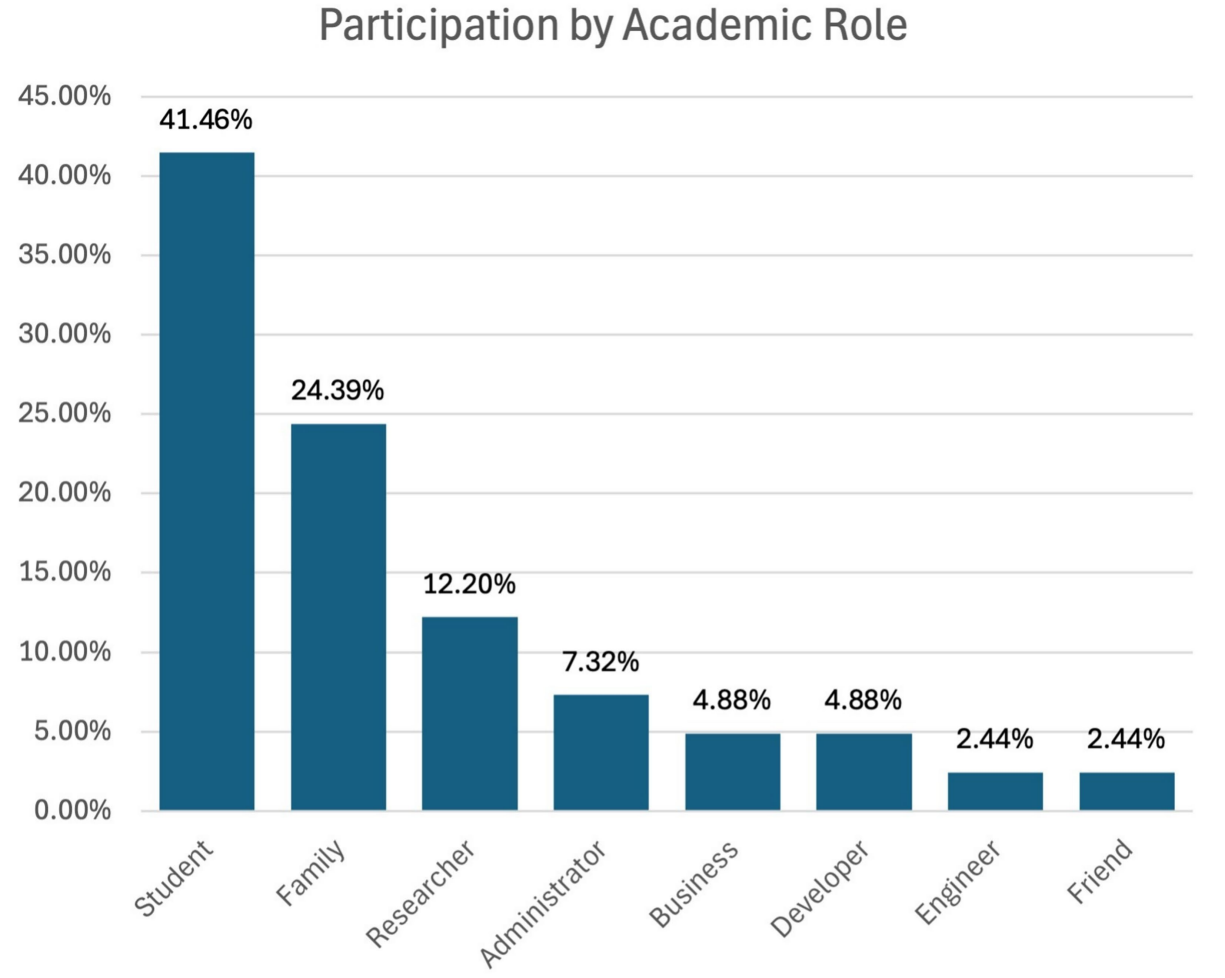
Respondents grouping by academic role.

The research contributors formed 12.20%, and the other party, made up of professionals plus members of the public, was 9.76%. The administrators were 7.32% and the developers 4.88%, as depicted in Figure 2.

### 4.3 Familiarity with LLMs

Among the respondents, 90% said they recognized Large Language Models, including ChatGPT, with 10% of the participants stating that they were not acquainted with the technology.

**Figure 3:**
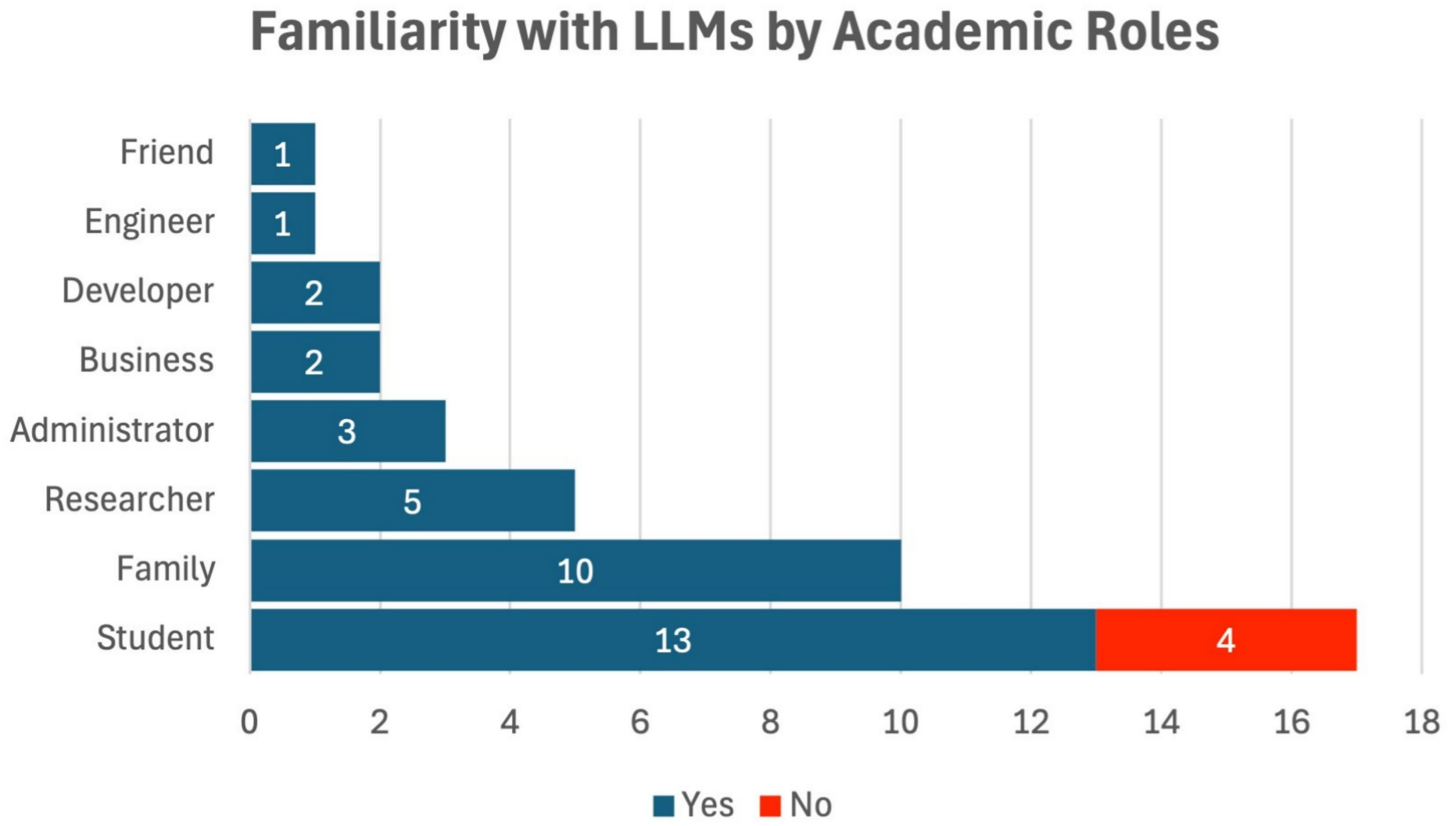
Respondents’ familiarity with LLMs by academic roles.

Of the 90% of the respondents who had heard of LLMs, 59.46% them were male, 37.84% were female, and 2.70% were in the non-support category in terms of gender. Regarding this, 23.53% of the participants admitted that they have no previous knowledge and experience with ChatGPT and other LLMs, which summed up students who have no idea about such technology at 100%, while 76.47% of students composed 31.71% of the yes responses to the query regarding their familiarity with ChatGPT and other LLMs. Figure 3 illustrates the respondents’ familiarity with Large Language Models (LLMs) by academic role, showing that 90% of respondents were familiar with LLMs. The majority of those familiar with the technology were students (76.47%), while faculty and administrators exhibited lower familiarity levels. This disparity suggests that students are the primary group engaging with LLMs, likely due to their direct involvement with tools like ChatGPT for academic tasks. The relatively low familiarity among faculty and administrators may indicate a lag in the adoption of such technologies at higher levels of academia, potentially reflecting a gap in training or awareness regarding AI’s capabilities and ethical challenges.

### 4.4 LLM usage for academic tasks

**Figure 4:**
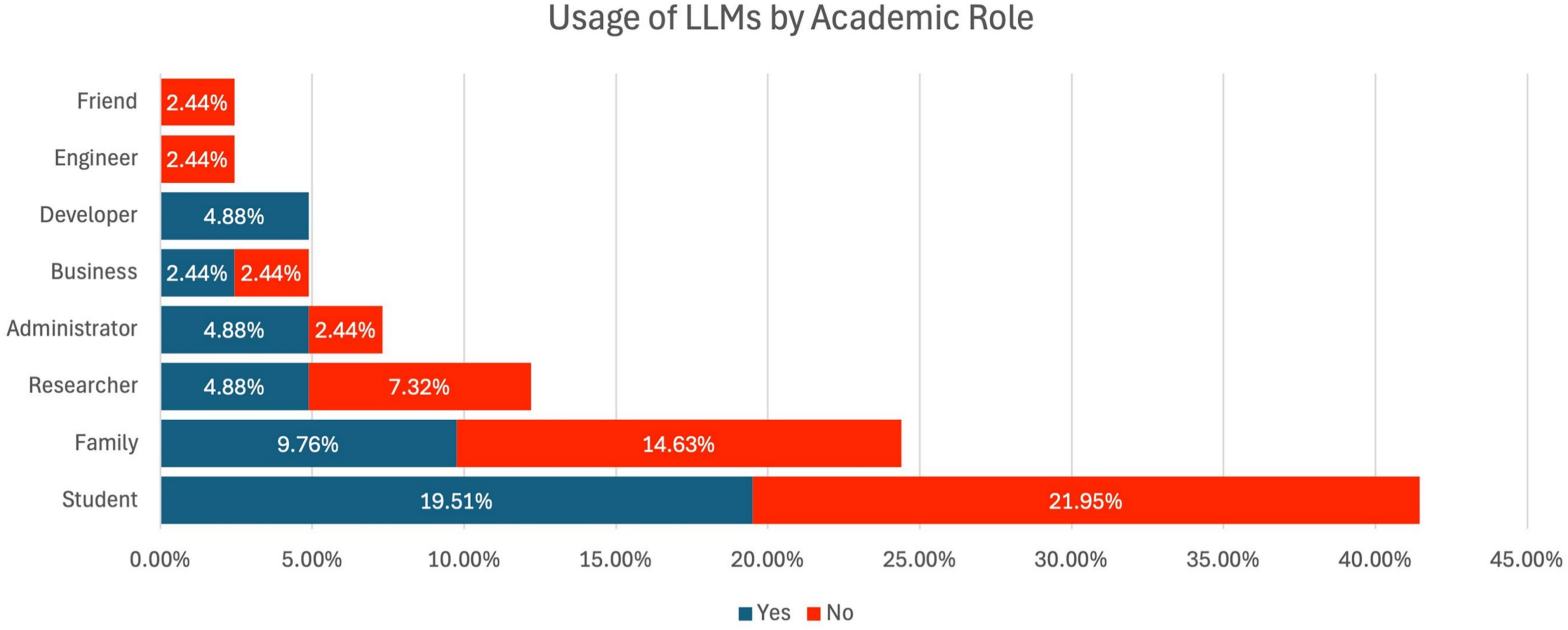
Respondents’ usage of LLMs by academic roles.

53.66% of the respondents indicated that they have never used LLMs in a personal context. This use could be any content generation, research assistance, or writing assistance. However, the largest number of respondents (46.34%) indicated that they had never used any of the Large Language Models. Figure 4 illustrates respondents’ usage of LLMs by academic roles. The higher usage rate of LLMs in academic contexts reflects the increasing integration of such technologies, though a significant portion of the academic population (46.34%) remains unfamiliar with or hesitant to adopt LLMs.

Out of all the respondents who admitted to having used LLMs for academic-related activities, 54.55% were male, 40.91% were female, and 4.55% said they did not want to state their gender. On the other hand, respondents who said no to using LLMs were 57.89% males, 36.84% females, and 5.26% unknown gender.

While comparing the different categories of participation in the usage of LLM and analyzing the utilization of LLM in terms of their academic tenure, a higher percentage of participants affirm their experience with the usage of LLM for academic tenure, except for the educational administrator. All the respondents of this survey stated that they had ever used LLMs for academic purposes if they were developers.

Figure 4 presents data on the usage of LLMs for academic purposes. While 53.66% of respondents reported using LLMs in personal contexts, only 46.34% indicated using them in academic settings. This finding aligns with the notion that while LLMs are seen as valuable tools for content generation and research assistance, their integration into formal academic work is still limited. The hesitation among respondents to use LLMs in academic contexts could be attributed to concerns about the ethical implications, such as plagiarism or academic integrity.

### 4.5 Awareness of ethical implications of LLMs in academics

**Figure 5:**
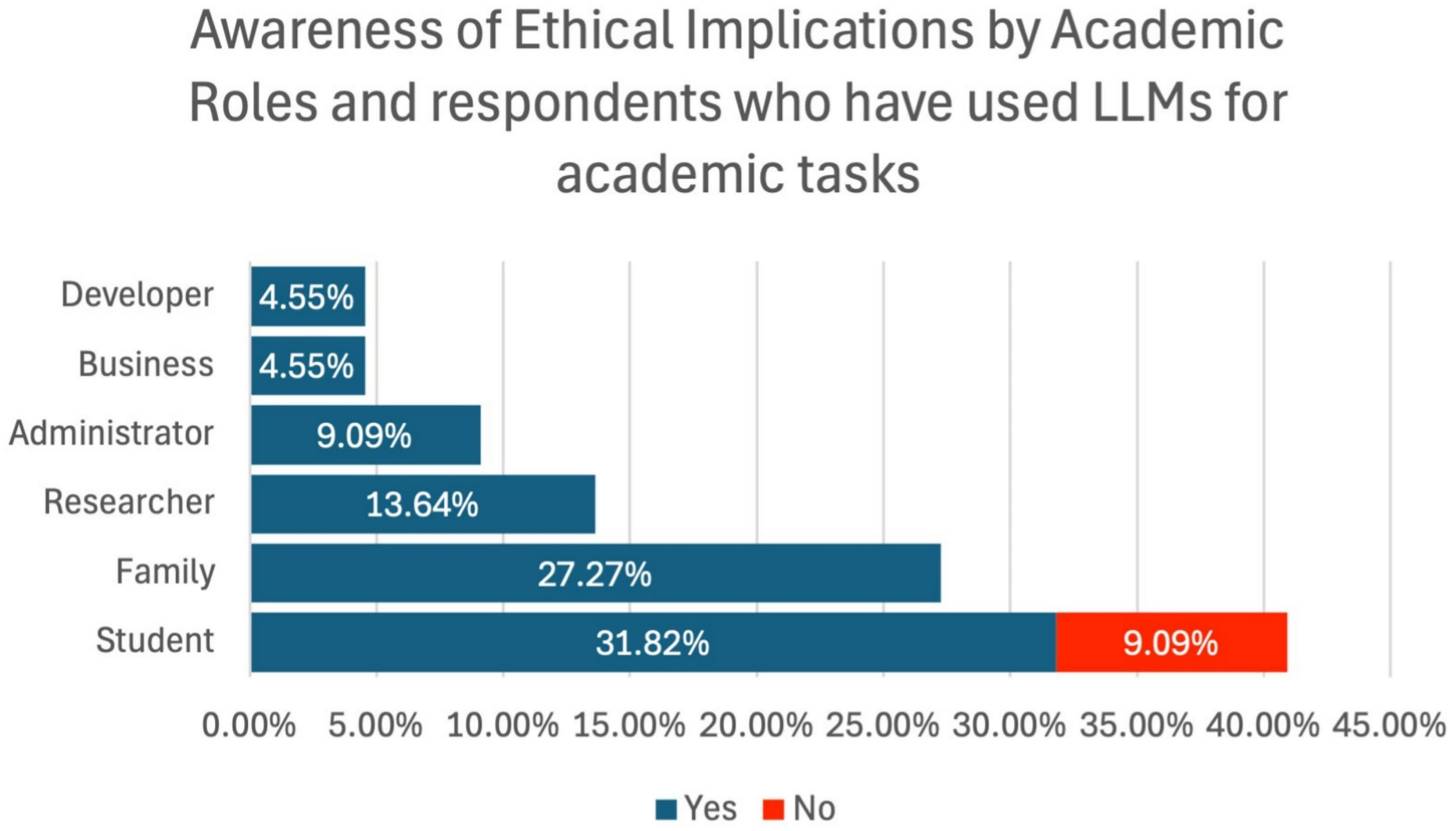
Respondents’ ethical implications awareness by academic roles.

Awareness of the ethical concerns related to the use of LLMs like ChatGPT in learning environments was established, with 90.91% of respondents affirming their awareness of the same, while 9.09% were unaware of the same. Figure 5 exhibits respondents’ ethical implications awareness by academic roles.

Figure 5 shows the level of awareness regarding the ethical implications of LLM usage in academia, segmented by the respondents’ academic roles. A clear majority (90.91%) of respondents expressed awareness of the ethical concerns associated with LLMs, including issues such as academic integrity, plagiarism, and data privacy. Only 9.09% were unaware of these ethical concerns. The figure highlights a high level of ethical consciousness across all academic roles, suggesting that while there is widespread recognition of the potential risks of LLMs, further educational efforts may be necessary, particularly to address the concerns of those less informed.

Out of the participants who have never used LLMs for academic purposes, there is a possibility that 9.09% of the users were ignorant of ethical concerns concerning the use of LLMs in an academic context.

### 4.6 Confidence in distinguishing AI vs. non-AI-generated work

Most of the respondents found it difficult to identify which work belonged to the AI category and which did not. As few as 5% were extremely confident while distinguishing between such categories.

**Figure 6:**
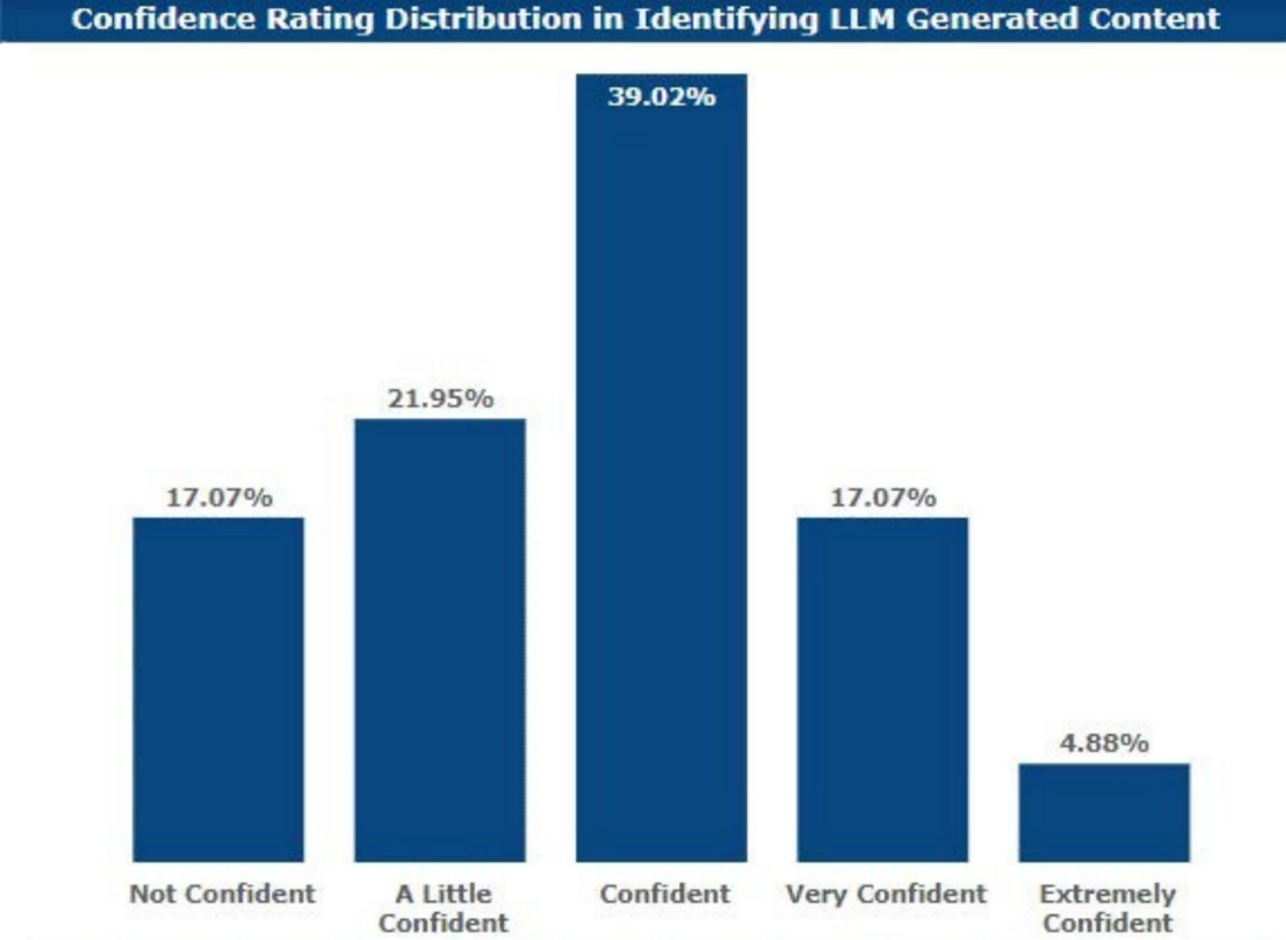
Confidence in AI-generated text.

Also, those who showed very high confidence were 17.07%; those who could be described as having high confidence were 39.02%; those with moderate confidence were 21.95%; and those who had low confidence or no confidence at all were 17.07%. Figure 6 maps out confidence in AI-generated text.

### 4.7 Content attribution

When asked about the relevance of crediting content produced by LLMs in academic work, 41.46% of the respondents said this is extremely important. Moreover, 17.07% of the respondents replied that it was very important, 26.83% said that it was important, 7.32% said that it was somewhat important, and 7.32% said that it was not important.

These responses were obtained when the content analysis was done using the academic roles, and all the faculty members gave their consensus on the extent of proper attribution of the content generated by LLM as important, very important, or extremely important. Concerning attribution, the majority of students understood its importance, as did most students in the previous questions. However, a few students negated its importance, stating that it was unimportant or of little importance.

**Figure 7:**
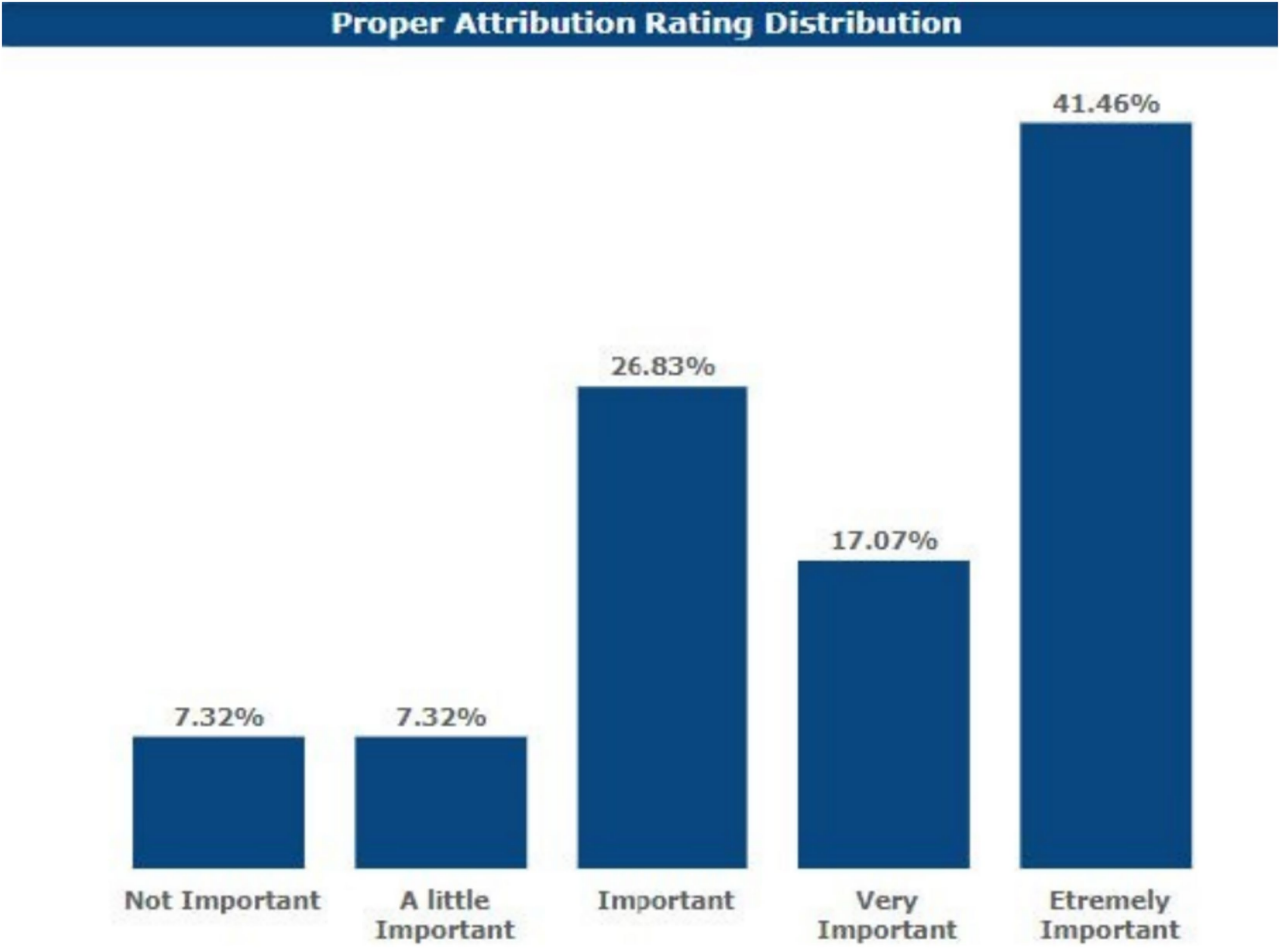
Respondents’ views on content attribution.

Additionally, Figure 7 shows that 41.46% of respondents consider content attribution for LLM-generated work extremely important. This highlights the growing recognition among academics of the need to maintain transparency and accountability in academic work. However, 7.32% of respondents who did not consider attribution important may reflect a lack of awareness or differing perspectives on AI’s role in academic contributions.

**Figure 8:**
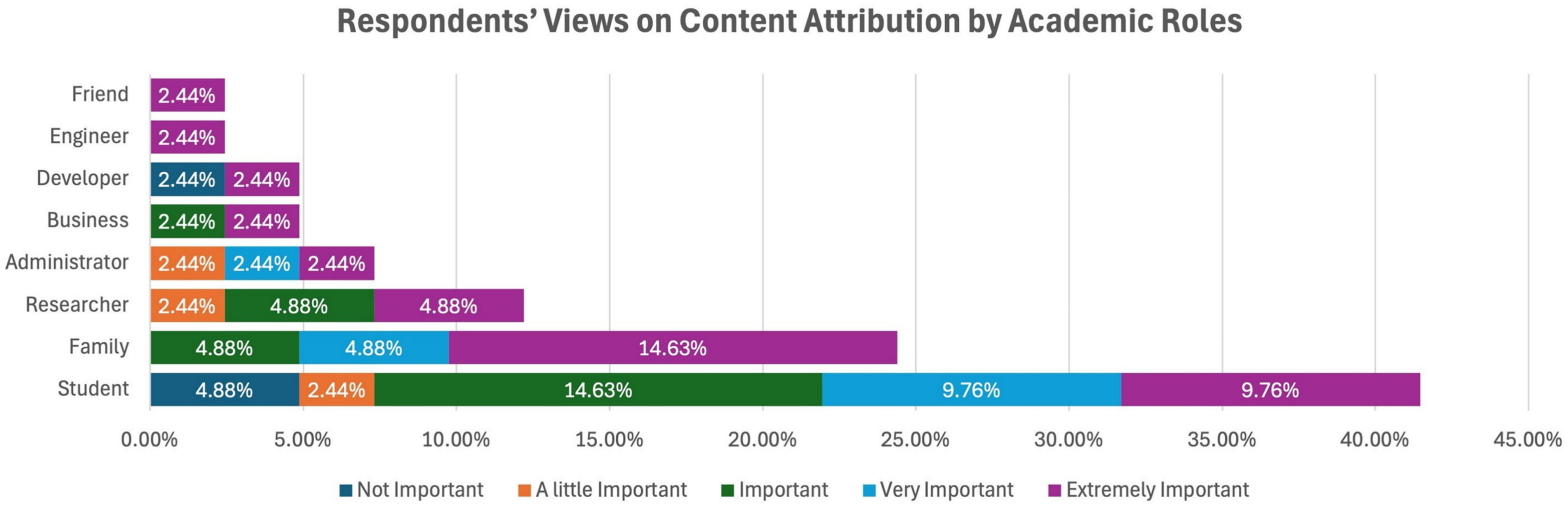
Respondents’ views on content attribution by academic roles.

Thirty-five percent of developers considered attribution unimportant, and the rest, or 35 %, saw it as extremely important. It emerged that for the heightened importance of attribution, both the researchers and administrators considered it either very important or slightly important. All other respondents considered it very/extremely important for proper attribution, either very important or extremely important (see Figure 8).

### 4.8 Convenience vs. ethical concerns

When participants were asked whether the convenience and the benefits of using LLMs in academia are worth the ethical drawbacks that came with their usage, 56.1% of the participants remained on the fence. This high percentage suggests that a significant portion of respondents are unsure about the balance between benefits and potential ethical drawbacks. Interestingly, 24.39% of participants prioritized ethical considerations over the convenience of LLMs, highlighting a substantial ethical divide in perceptions.

The frequency distribution by the roles of the academics and the proportion of academically engaged respondents showed diverse views on the relative appraisal of the convenience and efficiency benefits of LLM use against ethical concerns in academia. From these respondents, a large portion of students (25.71%), faculty and instructors (20%), and administrators (5.71%) believed that other considerations were needed to determine whether convenience and efficiency gains achievable with LLMs warranted the ethical considerations of using them.

**Figure 9:**
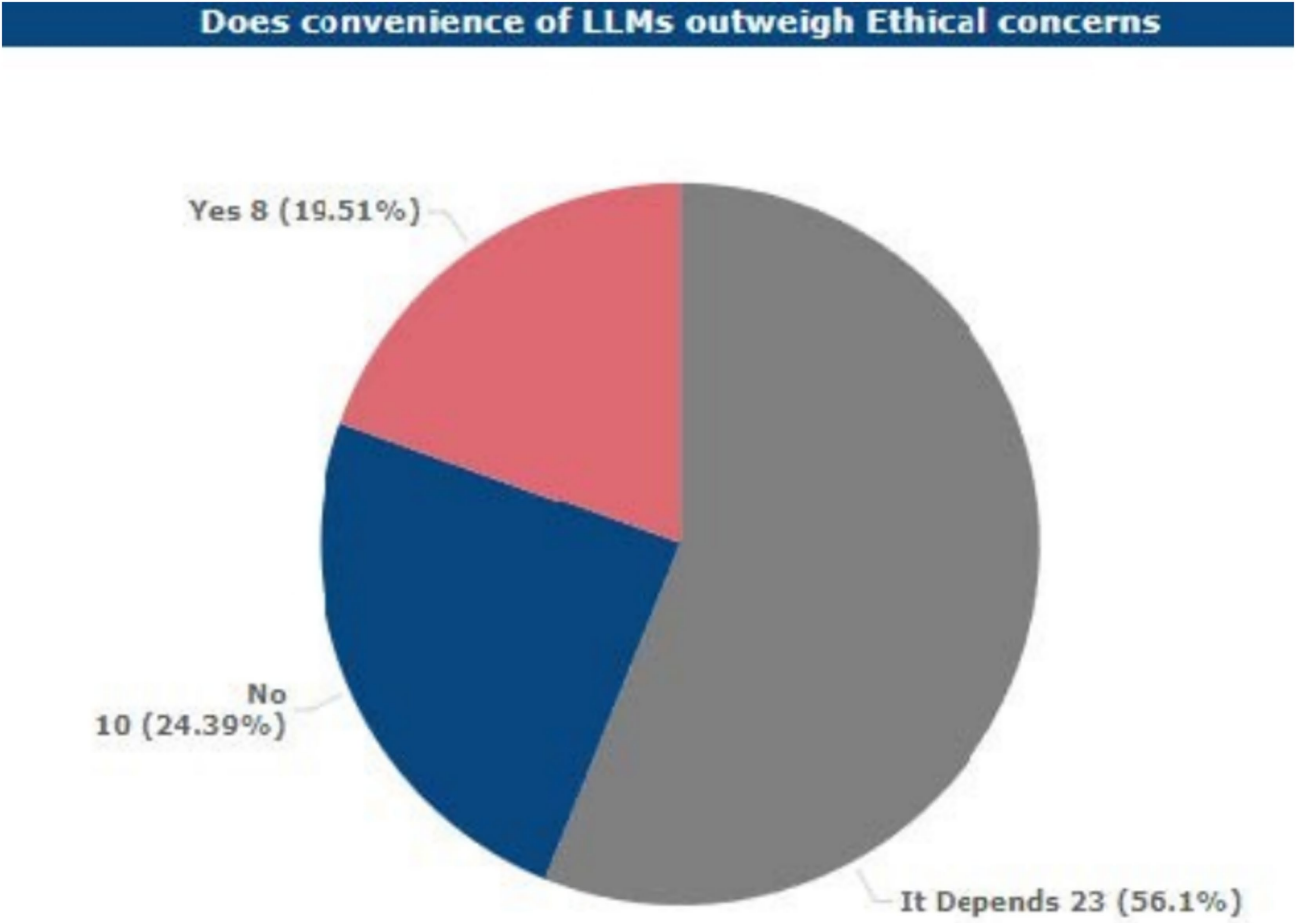
Respondents’ views on convenience of LLMs over ethical concerns.

Precisely, 11.43% of the students, 5.71% of the faculty members, and 2.86% of the administrators stated that overall, the advantages of using LLMs do more than the ethical issues. At the same time, 11.43% of students, 2.86% of faculty, and 8.57% of researchers stated that ethical issues should be regarded as more significant than the possible benefits of LLMs (see Figure 9).

### 4.9 Key ethical concerns on LLMs’ usage

Some of the ethical issues emerging out of this survey with regards to the integration of LLMs into academia include: The most serious risk mentioned by the participants is 18.75%, and it is related to the possible accrual of accidental academic property theft with the help of LLMs. The second most common concern stated was loss of originality of the contents generated by academics with the use of LLMs at 15.63%. With the use of LLMs in academia, 9.38% of users expressed the concern that the possibility of fraudulently using LLMs is not known. Some 7.81% of the respondents stated that ‘there is a bias in LLM outputs which is inherent, although they did not give further details as to what degree this was a concern. This same percentage, 7.81%, expressed their concern that students oversimplify their work by rushing to get LLM help. 7.81% of the respondents pointed out that LLMs may threaten academic integrity.

**Figure 10:**
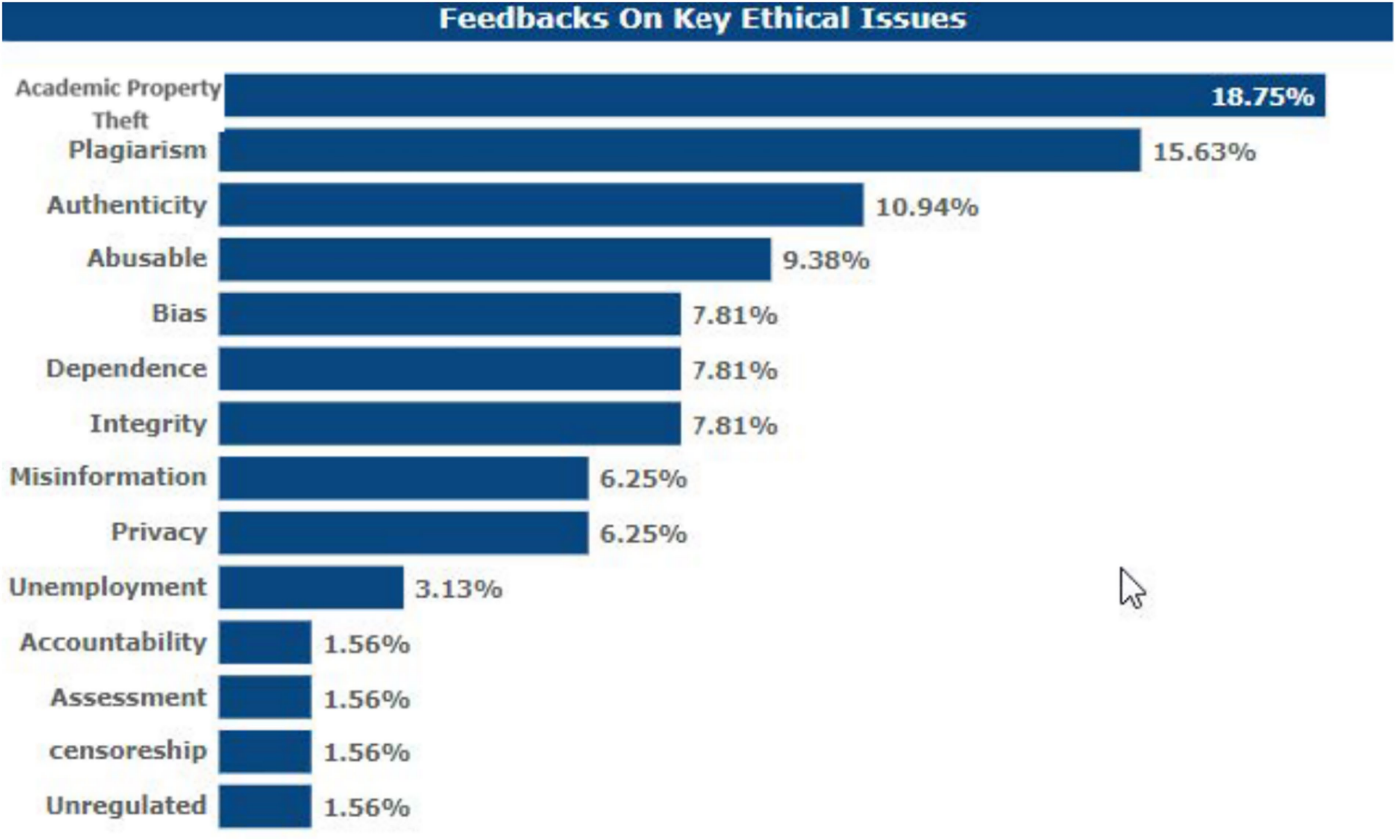
Respondents’ views on key ethical concerns.

6.25% said that LLMs would amplify false information. Data privacy issues related to LLM training were an issue of concern, as pointed out by 6.25% of the respondents. Another percentage (3.13%) expressed their concern about the rising unemployment levels upon implementation of the LLM integration. One of the issues that was raised and which I fully understood is the question of how one can make an AI responsible for its generated information, which was indicated by 1.56%. The ease of determining how much the students know when they use AI for assignments was a concern for 1.56%; 1.56% argued that LLMs are not sufficiently filtered to satisfy academic environments that may have people of different ages. The lack of regulations for LLM use was a concern to 1.56% of the respondents before contemplating including it in their academic needs (see Figure 10).

### 4.10 LLM predicted, adoption ratings, and sentiments

**Figure 11:**
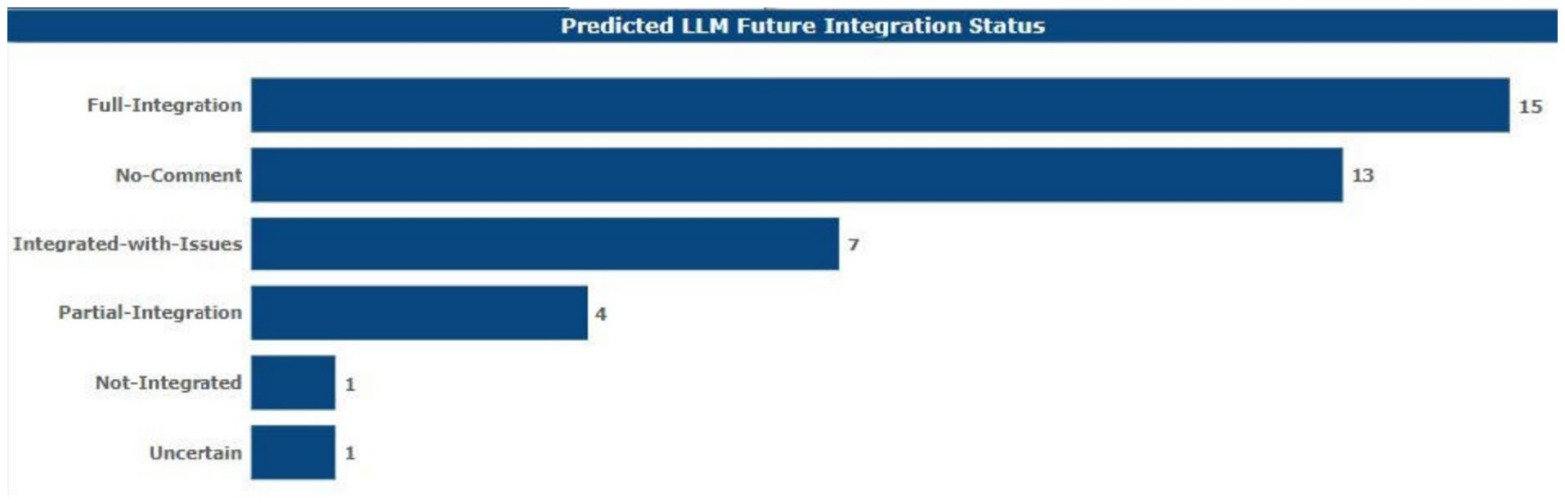
Future integration prediction.

Of all the respondents, 15 of them volunteered their forecast regarding the future place of LLMs in academia to be fully assimilated in the years to come. Thirteen said they would not respond. Seven of them expected that LLMs would be integrated regardless of the ethical issues pointed out above (see Figure 11).

Only one LLM specifically mentioned integration within the current and subsequent years, four other respondents anticipated that integration would only be partial, one respondent stated that he or she foresaw no integration at all in the academic sense of the term, and finally, one more respondent did not understand how some LLMs would integrate. In the same ratio of 63.41 and 36.59% of the total negative impression within the opinions of the respondents on the status of LLMs in academia over the next couple of years, there was a dominating negative perception.

More so, from the Supplemental Feedback Analysis, the respondents had a fair negative sentiment at 92.68% while the positive sentiment result was 7.32% only.

**Figure 12:**
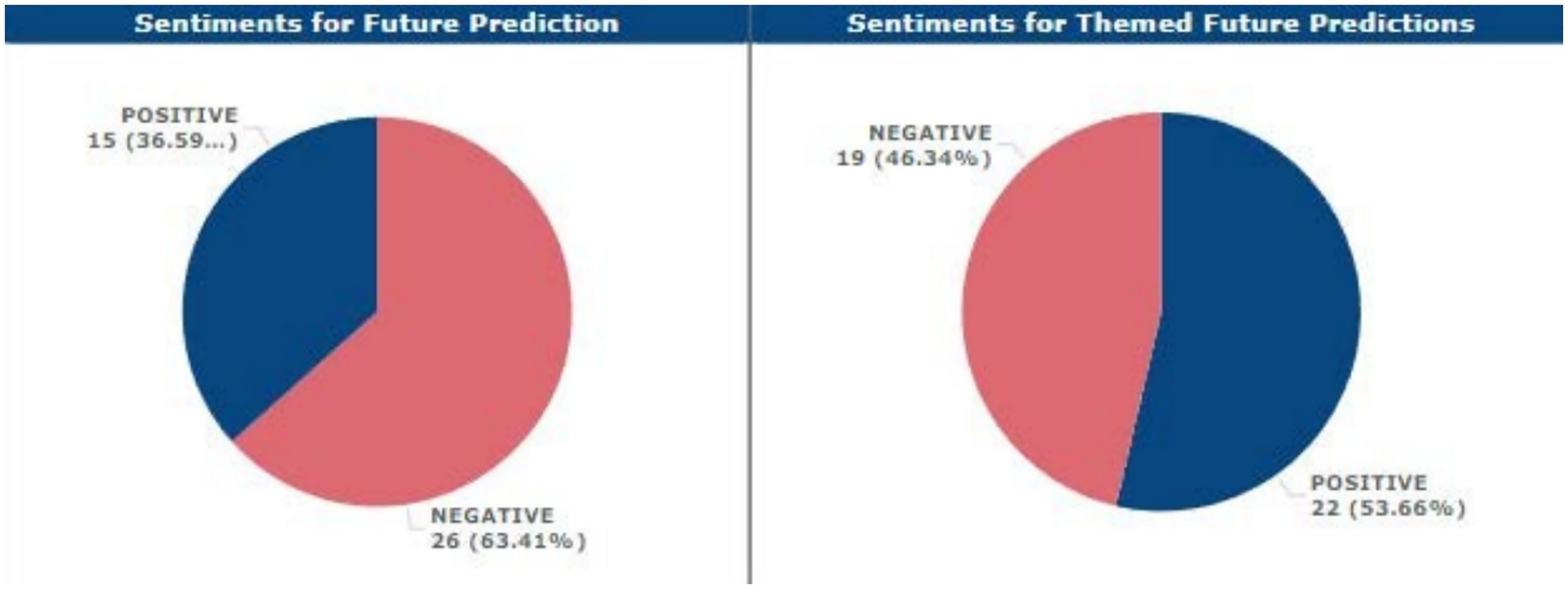
Sentimental analysis for future integration predictions, themed and unthemed.

**Figure 13:**
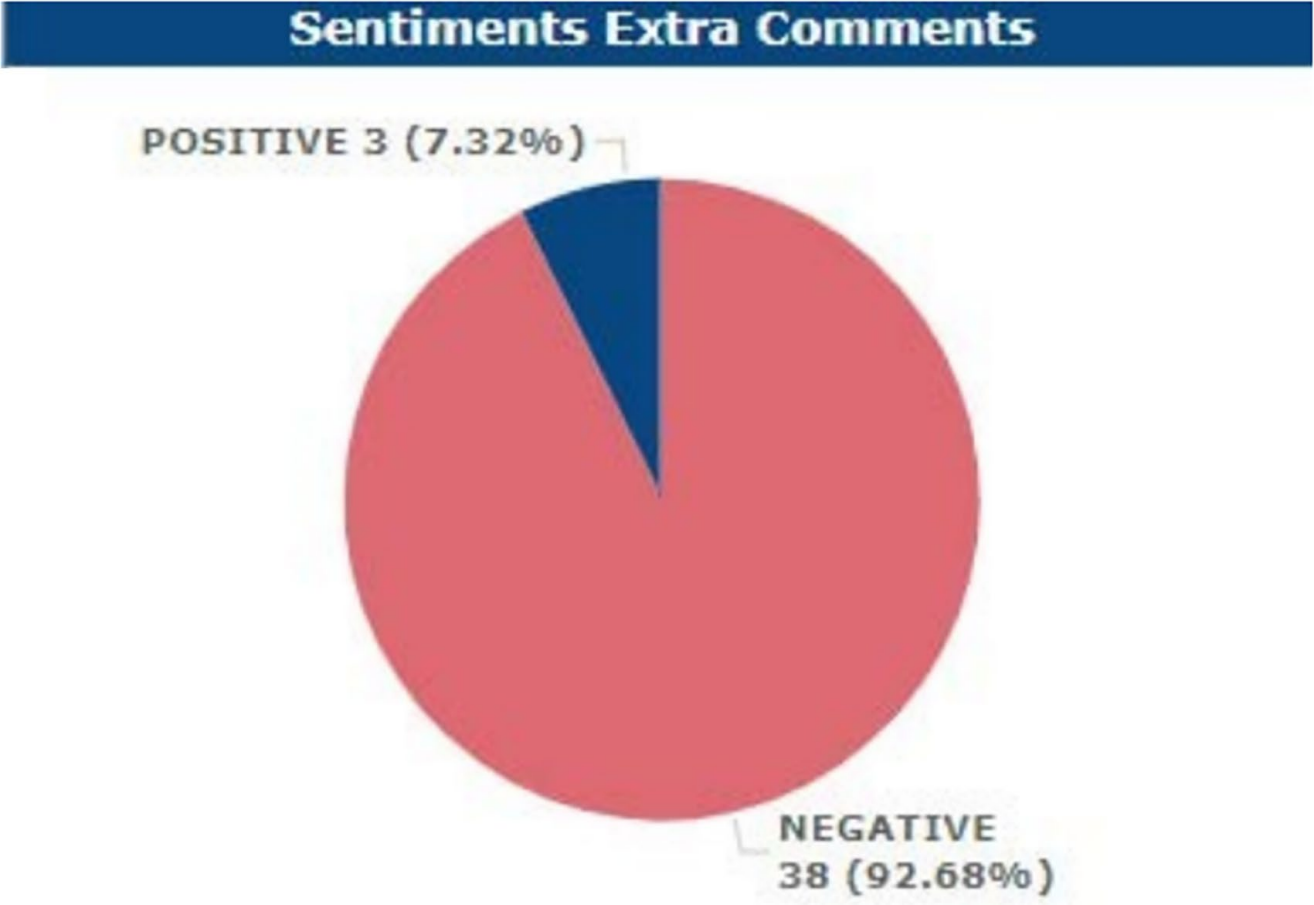
Sentimental analysis of extra comments.

While the data presented in the results section provides valuable quantitative insights, a deeper exploration of patterns, contradictions, and implications is needed. For example, the fact that 56.1% of respondents remained uncertain about the ethical implications of LLMs points to a significant ethical ambivalence. This uncertainty may suggest that while there is general awareness of technology, its complex ethical ramifications have not been sufficiently explored or addressed. The high level of ambivalence calls for further educational initiatives to help stakeholders navigate the ethical landscape of AI in academia (see Figures 12, 13).

Furthermore, the findings raise several contradictions. While 41.46% of respondents believe content attribution for LLM-generated work is extremely important, there remains a notable minority (7.32%) who consider it unimportant. This divergence in opinions suggests a need for clearer guidelines and training on best practices for AI content attribution, particularly as LLMs become more integrated into academic workflows.

## 5 Discussions

This work gathers the views of academic stakeholders and analyzes the literature to create a set of ethical standards, specifically for academia, when implementing LLMs. Our objectives were to look at the literature, identify stakeholder perceptions, and provide adoption recommendations.

### 5.1 Stakeholders’ viewpoints

There is high LLM familiarity among stakeholders, with 90% of them familiar with LLMs, as 30% of students used ChatGPT in 2022, according to Saúde et al. (2024). But 9.09% of these users did not know about the ethical issues; therefore, there need for sensitization on the appropriate use of AI. Besides, 70.73% of respondents associated LLMs with accidental plagiarism.

Nonetheless, as the LLM is widely used, only 17.07% of the students appreciate the content generated by the tool as unique. An emphasis is placed on convenience, with 56.1% of participants noting that they balance ethical and practical considerations, while 19.51% of participants marked convenience over ethics, and 24.39% responded by choosing ethical considerations over the convenience of use.

### 5.2 Pedagogical dynamics and LLMs

As Crompton and Burke (2023) also observes, respondents have a very low level of confidence in identifying human work from LLM output of only 4.88%. The uncertainty favors 95.22% of the stakeholders, increasing the tendency of underemphasizing human authorship. In his article of 2023, OpenAI noted that LLMs are being increasingly used in teaching, although educators have expressed concern about plagiarism. It is suggested that institutions should develop adequate policies to counter ethical problems and pessimistic attitudes toward LLM usage.

### 5.3 Creating best practices for LLM programs

1. Create Attribution Standards

The approaches to the identification of LLM must be clear to avoid compromising the academic standards. Consequently, the institutions should use tools that can detect LLM-generated content within their academic institutions. In as much as watermarking may not be appropriate for every professional environment, it is appropriate in academia to maintain an enduring display of the watermark.

2. Regulation, reform, and monitor

Therefore, regulation is the way forward in ensuring that the use of LLM fits the ethical standards. A similar mechanism should be put in place that enables various institutions to ensure responsible monitoring and regulation of curricula for LLMs and satisfactory stakeholder assurance.

3. The LLM must have awareness programs

Mandatory courses are training students and faculty to appraise what LLMs can do, the moral implications of their use, and how they should be used positively. All these programs must be periodically revised due to progress in AI and provide academic communities with the tools required for the ethical implementation of LLMs.

### 5.4 Limitations of the study

With non-probability sampling, it is hard to generalize the results; combining both qualitative and quantitative data can also bias the results. Furthermore, the fact is that AI advances relatively quickly and, thus, some of the findings can quickly become obsolete. The use of self-reported data may introduce potential biases whose effects would expand the gap between this study’s findings and findings easily relatable to the broader academic fraternity.

## 6 Conclusions and recommendations

This study provides a foundational understanding of the ethical challenges posed by using Large Language Models (LLMs), such as ChatGPT, in educational contexts. By centring stakeholder perceptions—spanning students, educators, administrators, and AI developers, the research identifies critical concerns, including plagiarism, authenticity, bias, misinformation, and privacy. While these issues align with concerns previously raised in the literature, this study further highlights the pressing need for structured institutional responses to ensure responsible and ethical LLM integration.

### 6.1 Key findings

- Widespread adoption of LLMs in academic settings has outpaced the development of formal ethical guidelines.
- Stakeholders express significant concern over the misuse of LLMs, particularly around plagiarism, bias in content, and a lack of attribution standards.
- There is a clear demand for institutional policies and practical frameworks to guide ethical LLM usage.

### 6.2 Recommendations for ethical integration

To address these concerns, the study proposes the following concrete, implementable strategies:

1. Development of Clear Ethical Guidelines:

- Institutions should establish and disseminate concise, accessible, and adaptable LLM usage policies.
- Guidelines should address issues of academic integrity, clarify acceptable uses of LLMs, and define AI-assisted vs. AI-generated content.

2. Mandatory Training and Awareness Programs:

- Introduce training sessions for students, faculty, and staff to enhance digital and AI literacy.
- These programs should include modules on ethical risks (e.g., bias, misinformation), responsible authorship, and proper citation of AI tools.

3. Attribution Standards and Citation Practices:

- Develop and implement standard protocols for acknowledging AI-generated content in academic work.
- Institutions can adopt or adapt existing citation formats (e.g., APA, MLA) to explicitly reference LLMs.

4. Implementation of Institutional AI Review Panels:

- Create cross-functional ethics committees to assess and revise LLM usage policies regularly.
- These panels should include representatives from academic leadership, IT, legal, and pedagogical teams.

5. Privacy Protection and Data Governance:

- Ensure any interaction with LLMs complies with data protection laws and institutional privacy policies.
- Educators and students must be made aware of how data shared with LLM platforms is stored and used.

6. Bias and Misinformation Mitigation Measures:

- Encourage the use of LLMs as supportive tools rather than sources of factual authority.
- Promote critical thinking and source verification as part of AI-assisted research practices.

7. Development and Use of AI-Detection Tools:

- Invest in AI-detection software to identify potential misuse or uncredited AI-generated work.
- Use such tools judiciously, ensuring they support them rather than police academic activities.

8. Cost-Effective and Scalable Solutions:

- Design frameworks that are budget-friendly and adaptable to different institutional sizes and capacities.
- Emphasize open-access tools and shared community resources where possible.

### 6.3 Future research directions

Further studies should focus on:

- Evaluating the real-world effectiveness of these guidelines in diverse academic contexts.
- Investigating students’ and educators’ behavioral responses to LLM regulations.
- Exploring long-term implications of LLM integration on pedagogy, assessment, and academic values.

This research contributes a pragmatic roadmap for the ethical integration of LLMs in academia, advocating for innovation without compromising academic integrity. By fostering continuous dialogue and refining ethical frameworks in step with technological advances, educational institutions can harness the benefits of LLMs while upholding the highest standards of scholarly conduct.

## Data Availability

The original contributions presented in the study are included in the article/supplementary material, further inquiries can be directed to the corresponding author.
